# The impact of olfactory loss on quality of life: a 2025 review

**DOI:** 10.1093/chemse/bjaf023

**Published:** 2025-07-25

**Authors:** Anna Oleszkiewicz, Ilona Croy, Thomas Hummel

**Affiliations:** Interdisciplinary Center Smell & Taste, Department of Otorhinolaryngology, Faculty of Medicine Carl Gustav Carus, Technische Universität Dresden, Dresden, Germany; Institute of Psychology, University of Wroclaw, Wrocław, Poland; Department of Clinical Psychology, Institute of Psychology, Friedrich-Schiller-University Jena, Jena, 07743 , Germany; Department of Psychotherapy and Psychosomatic Medicine, Faculty of Medicine and University Hospital Carl Gustav Carus, TUD Dresden University of Technology, Dresden, 01307, Germany; German Centre for Mental Health (DZPG), site Halle-Jena-Magdeburg, Halle-Jena-Magdeburg, Germany; Interdisciplinary Center Smell & Taste, Department of Otorhinolaryngology, Faculty of Medicine Carl Gustav Carus, Technische Universität Dresden, Dresden, Germany

**Keywords:** olfaction, olfactory loss, anosmia, mental health, coping strategies

## Abstract

For a long time, the sense of smell was considered the neglected stepbrother of human sensory abilities, and the loss of smell has received little attention. This perception changed dramatically with the COVID-19 pandemic, which led to millions of people losing their sense of smell, and some never recovering. COVID-19 not only increased general awareness of olfactory disorders but also accelerated research into the role of smell in nonverbal communication and mental health. This review aims to summarize the literature on the impact of olfactory disorders on quality of life. Starting from the functions of olfaction in healthy individuals, we will briefly describe the most common olfactory disorders and their effect on an individual’s life, including nutrition and eating behaviors, social and psychological well-being, and exposure to environmental hazards. Consequences of olfactory loss permeate many spheres of daily life. On average, dysosmia has a moderate impact on quality of life, though for some patients the effects can be severe.

## Introduction

The lay view on olfaction is that our sense of smell is not essential. This assumption was potentially based on older research indicating that humans are “microsmic” (Loos et al. 2023; McGann 2017) and surveys reporting that people declare they would rather give up their sense of smell than any of their other four main senses (Schifferstein 2006), their smartphones (McCann Worldgroup 2011), their little toe, or hearing in one ear (Wrzesniewski 1999). However, the scientific view on the capacity and functions of olfaction for humans changed dramatically in the last decades (McGann 2017; Loos et al. 2023), and general awareness increased due to the COVID-19-related smell loss that affected millions of people (Desiato et al. 2021).

## Olfaction—one of the three chemical senses

Olfaction is one of the sensory systems serving to detect and decode chemical stimuli in the environment. In olfaction, volatile chemical compounds in the air are inhaled through the nose, bind to olfactory receptors located in the olfactory epithelium, and generate signals that are integrated by the olfactory bulb before being transmitted to the primary and secondary olfactory cortices for interpretation. Gustation allows detection and perception of the chemical compounds dissolved in saliva to give the sensations of salty, sweet, sour, bitter, or umami tastes, possibly also fat and water. Chemesthesis allows detection of agents that have reached the mucosa (for instance of the oral and nasal cavity) by activating receptors involved in other sensory systems, to raise sensations such as burning, stinging, pain, stringency, or cooling. All three senses interact in aroma perception during eating.

## Functions of olfaction

As comprehensively delineated by Stevenson (2010), the main functions of olfaction include guidance toward objects with positive connotations, such as delicious foods or beautifully smelling flowers, cueing social interactions, and warning about environmental hazards like spoiled food or leaking gas. The individual hedonic valence of an odor hence determines the behavioral response of approaching or avoiding the source of a smell (Arshamian et al. 2017).

Olfaction is a key modality for regulating appetite, food intake, and appreciation of a meal. Pleasantly perceived food odors indicate edibility, increase appetite, and aid food localization. During chewing, odorous molecules are pumped into the retronasal passage and reach the olfactory mucosa, evoking olfactory perception of food aroma (Boesveldt and de Graaf 2017). Thus, both orthonasal and retronasal routes bear a regulatory role in eating behaviors.

Olfaction is also a cue in social communication. Body odors transport a variety of information guiding nonverbal communication, such as familiarity, nutrition, hormonal status, emotionality, or inflammation (Loos et al. 2023). As a result, body odors impact mother–child bonding (Schäfer and Croy 2023) and mating (Mahmut and Croy 2019).

Concerning the olfactory warning function, olfaction is a rather slow processing near-distance sense. Human olfaction appears to be tuned to detect gaseous hazards at very low concentrations, such as smoke and fire but also pathogens or metabolic products of pathogens, as they evolve in inflamed organisms or decay, for instance. Those odors evoke a typical disgusted expression (Saluja et al. 2024).

Situations in which we use the sense of smell are almost always multimodal processes involving multiple sensory modalities, such as tasting, smelling, and chemesthesis for eating, or seeing, hearing, and smelling for person perception. Olfaction facilitates spatial navigation and the formation of cognitive maps (Raithel and Gottfried 2021; Schwarz et al. 2024). It also supports some motor functions like mobility, balance, fine motor function, manual dexterity (Tian et al. 2016), respiratory function (Gorodisky et al. 2024), and swallowing (Ryan and Hummel 2013; Jestrović et al. 2015; Loret 2015; Yamamura et al. 2016).

## Types of olfactory impairment and prevalence

### Olfactory loss etiologies

Many people gradually lose their sense of smell, with aging being the main cause of olfactory deterioration (Doty and Kamath 2014). Other causes for gradual olfactory loss include allergic rhinitis, chronic rhinosinusitis, or neurodegenerative disorders (Whitcroft et al. 2023). Idiopathic olfactory loss (without a clear underlying cause) is associated with an increased risk of developing neurodegenerative diseases such as Parkinson’s disease (Doty et al. 1988; Ponsen et al. 2004, 2009; Haehner et al. 2009, 2011), Alzheimer’s disease (Thomann et al. 2009; Wilson et al. 2009), or dementia (McLaughlin and Westervelt 2008; Stanciu et al. 2014). However, some people are congenitally anosmic, meaning they were born without a sense of smell. Congenital anosmia is relatively rare (Karstensen and Tommerup 2012; Schriever and Hummel 2020). It often has a genetic basis and can be an associated symptom of syndromes, such as Kallman syndrome, CHARGE syndrome, the Bardet-Biedl syndrome, and SCN9A-associated insensitivity to pain (Deller et al. 2022).

### Quantitative olfactory impairment

Screening tests such as *the* “University of Pennsylvania Smell Identification Test” (UPSIT) (Doty et al. 1984) or the “Sniffin’ Sticks” (Hummel et al. 1997) can reliably distinguish olfactory impairment from normal olfactory function based on an individual numerical score referred to normative values in the population. Individual scores can be categorized as normosmic (normal olfactory function), hyposmic (impaired olfactory function), or anosmic (including a residual ability to perceive odors with limited usefulness in daily life or no olfactory function). Exceptionally high scores are termed hyperosmia (Hernandez et al. 2023).

### Qualitative olfactory impairment

Patients may also experience qualitative olfactory disorders. Parosmia is a distorted olfactory sensation in the presence of an odor source (e.g. sensing coffee aroma as a rotten smell). Phantosmia is an olfactory sensation without the presence of an odor source, i.e. olfactory hallucination (Frasnelli et al. 2004). Qualitative olfactory dysfunction typically manifests with unpleasant olfactory perceptions. Patients with parosmia complain that coffee, meat, onion, or toothpaste begin to smell repulsive and disgusting. Some patients also complain about triggered, identifiable, and usually unpleasant olfactory percepts that persist, sometimes for days, in the absence of an ongoing stimulus, what has been called “odor-induced phantosmias.” Qualitative olfactory disorders are often symptoms accompanying recovery from a viral infection, possibly due to a miswiring of newly proliferating olfactory receptor neurons in the olfactory bulb but have also been reported by patients with sinonasal and post-traumatic olfactory dysfunction (Pellegrino et al. 2021).

**Fig. 1.**
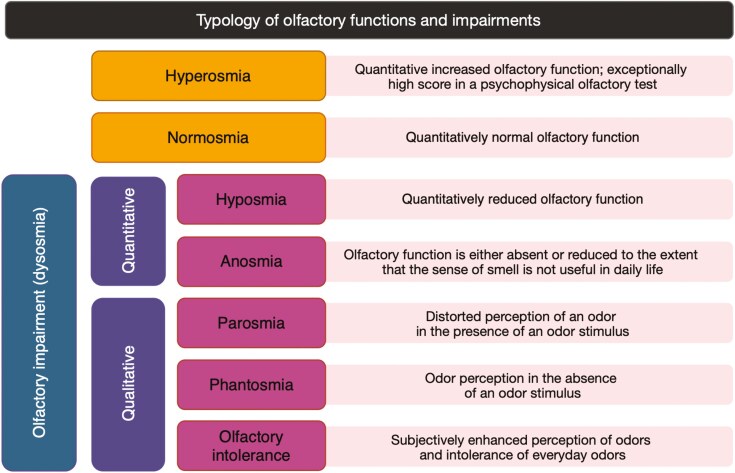
Causes and types of olfactory impairment.

A distinctive category of qualitative olfactory impairment is complaints about the subjectively enhanced olfactory perception that is termed “chemical odor intolerance.” Odor intolerance manifests with the experience of feeling ill (e.g. nausea, headache, breathing difficulties) in the presence of ambient everyday odors (Hernandez et al. 2023; Nordin Millqvist, et al. 2003; Ryan et al. 2006). The types of olfactory functions and impairments are summarized in Fig. 1.

### Olfactory loss prevalence

A meta-analysis summarizing results from 25 epidemiological studies, including a population of 175,073 participants aged between 18 and 101 years (mean age 63 years, 56% men), indicates the overall prevalence of olfactory disorders to be 22.2% (95% CI 14.8 to 30.6%). Based on the results of psychophysical tests, the prevalence is 28.8%, and based on the self-reported olfactory disorders, it is 9.5% (Desiato et al. 2021). Other studies suggest that anosmia concerns 3.6% to 5.8% of the general population, while hyposmia is estimated to be present in 13% to 18% of people (Hummel et al. 2017). Parosmia occurs in 4.8% of the general population (Olofsson et al. 2022), and phantosmia in 4.2% (Wehling et al. 2021). Qualitative olfactory disorders are often (19% to 34%) comorbid with quantitative olfactory dysfunction, i.e. hyposmia or anosmia (Reden et al. 2006; Pellegrino et al. 2021).

The gap between the self-reported olfactory loss and the results of psychophysical diagnostic tests (Desiato et al. 2021) indicates that many people have a distorted sense of smell but still consider their olfaction normal. Studies on the quality of life in patients with olfactory loss are often biased as the participants of these studies reported themselves to the ENT clinics. They decided to seek medical help because olfactory loss has become bothersome, and they want to eliminate it from their lives. But, since olfaction is the least valued of the senses, some people likely do not realize they are missing out on odors. Indeed, empirical evidence shows that there are people in the general population who rate their sense of smell as normal, while psychophysical tests suggest anosmia or hyposmia (Oleszkiewicz and Hummel, 2019), and for whom the lack of odor sensation is not distressing (Oleszkiewicz et al. 2019).

Epidemiological estimates for olfactory dysfunctions vary as a function of sample demographics, the definition of impairment, and measurement methods. Olfactory deficits are more frequent among men (Sorokowski et al. 2019) and the elderly (Attems et al. 2015; Olofsson et al. 2021). Previous COVID-19 infection also increases the odds of olfactory dysfunction, as 5% to 10% of the patients with COVID-19-associated olfactory loss do not fully recover (Kim et al. 2024).

### Consequences of olfactory loss in daily life

**Fig. 2.**
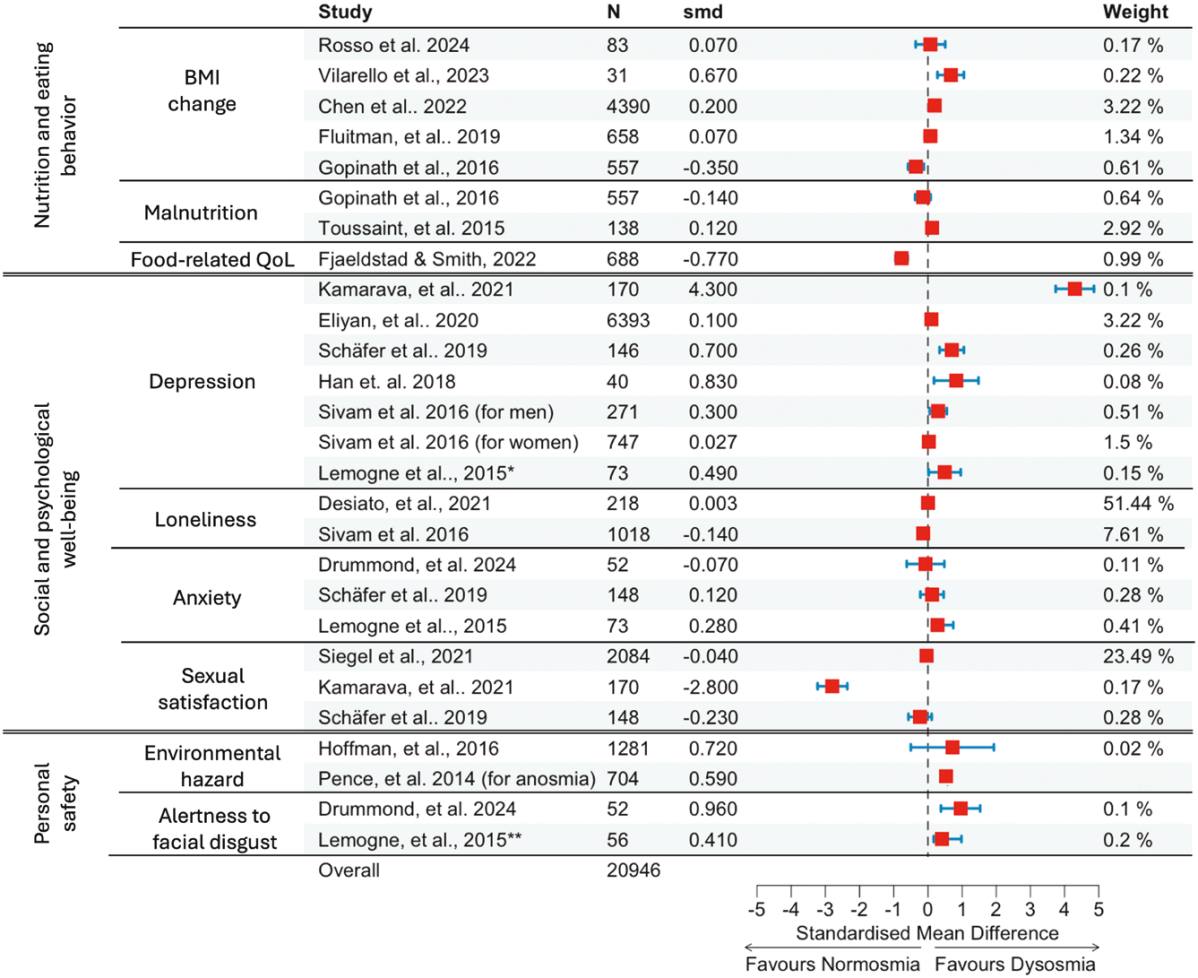
Forest plot of the effects of olfactory loss on the aspects of quality of life. The selection of studies for Fig. 2 was based on the following criteria: (1) published after 2014, (2) presenting a cross-sectional comparison of dysosmic (anosmic, hyposmic, or both) and healthy controls in the respected area impacted by the smell loss or longitudinal data for dysosmia, and (3) available descriptive data to calculate Cohen’s d effect size. Thus, the presented selection is not a systematic review or meta-analysis. Despite Kamarava (2021) study being an outlier, we decided not to exclude it from the Figure. Smd—Standardized mean difference (Cohen’s d), N—total number of participants for the study, QoL—quality of life. Horizontal blue lines depict 95% confidence interval for the effect sizes marked with red squares. Study weight is proportional to study precision, specifically the span of the standard error of the estimate of a study. For Pence et al. 2014 no confidence interval data is reported.

Patients with olfactory loss report complaints in the areas that mirror the functions of olfaction, i.e. nutrition, social and psychological functioning, and exposure to environmental hazards. We elaborate on these difficulties below and summarize the effect sizes for these complaints reported by the respective studies in Fig. 2. According to the effect sizes mentioned in the papers published in the last decade, the consequences of olfactory loss permeate many spheres of daily life, but this impact is not debilitating.

### Consequences for nutrition and eating behavior

#### Anosmia and hyposmia

Food odors trigger appetite and drive eating behaviors (Zoon et al. 2014; Morquecho-Campos et al. 2020). In patients with olfactory loss, retronasal olfaction has been found to contribute more to the quality of life than orthonasal olfaction, pinpointing the critical role of olfaction in food appreciation (Oleszkiewicz et al. 2019). Smell loss often has negative consequences on food intake and energy balance, leading to non-uniform weight changes. When food becomes flavorless, some patients suffering from smell loss lose interest in eating and reduce food consumption. Some are afraid of consuming rotten food because they are unable to assess food freshness accurately (BurgesWatson et al. 2021). These changes in eating habits may cause weight loss (Purdy et al. 2020) and malnutrition, the latter being prevalent in the geriatric population (Gopinath et al. 2016; Fluitman et al. 2021). Other patients with smell loss may gain weight by increasing food intake and consuming more sugary and fatty products to compensate for the lack of aroma perception (Gaillet-Torrent et al. 2014; Ramaekers et al. 2015; Proserpio et al. 2017; Van Regemorter et al. 2020; Chen et al. 2022; Vilarello et al. 2023; Rosso et al. 2025). Some studies yield no significant results on the change of weight in relation to olfactory loss (Fluitman et al. 2019), likely because the two mechanisms cancel each other out.

Eating is a social act, e.g. family and friends gathering, preparing meals together, and going out to restaurants, cafes, or bars. Olfactory loss deprives patients of active participation in these activities (Boesveldt and Parma, 2021), resulting in distress and the feeling of longing and depression (Burges Watson et al. 2018, 2021). With olfactory loss, food-related quality of life decreases. Eating becomes sustenance stripped of its joy. Patients with anosmia report avoiding cooking. Their decisions related to food choices become more complicated, confidence in their own cooking skills weakens, and the results of cooking become less predictable. Consequently, patients begin to see cooking as not a fulfilling activity and point to the inability to prepare new meals successfully (Fjaeldstad and Smith, 2022).

Coping strategies to appreciate food and drinks include a shift toward texture-rich foods to increase oral sensations (Burges Watson et al. 2021). The use of capsaicin, an active component of chilli peppers that produces a sensation of burning, may help patients with olfactory loss to increase the perception (and decrease the use) of salt (Hunter et al. 2023). An important, already progressing initiative is culinary education tailored for individuals suffering from olfactory disorders. People suffering from smell loss are instructed on how to make their food more interesting and stimulating while keeping it healthy and nutritious. The course-related cookbook presents recipes for texture-rich meals at varying temperatures (Fjaeldstad, 2024). Patients with anosmia are also known to develop compensatory mechanisms to aid in the lack of food-related chemosensory perceptions. It has been demonstrated that they better hear the level of liquid carbonization (providing more rich, trigeminal sensation) as compared to individuals with a normal sense of smell (Oleszkiewicz et al. 2023).

#### Parosmia and phantosmia

Parosmia and phantosmia have rather uniform consequences on eating behaviors, as the patients most often experience negative distortions of aroma perceptions. They describe food-related odorous and mouth sensations as “disgusting to eat”, “awful,” and “metallic”, resulting in food avoidance (Fjaeldstad and Smith, 2022). Some patients with parosmia mention leaving the house while the meal is being prepared to avoid repulsive olfactory sensations (Burges Watson et al. 2021; Pellegrino et al. 2021), often arising when roasting or heating (Fjaeldstad and Smith, 2022).

### Consequences for social and psychological well-being

#### Anosmia and hyposmia

Olfactory disorders are accompanied by depressive symptoms in 36% to 76% of the patients, depending on the etiology of the olfactory loss (Deems et al. 1991; Temmel et al. 2002a; Jung et al. 2014; Smith and Alt 2020; Kamrava et al. 2021; Sabiniewicz et al. 2022), in a dose-dependent way (Sharma et al. 2022a). Patients with olfactory loss present reduced central processing of emotional stimuli (Han et al. 2019). In congenital anosmia, 29% of the cases are associated with mild or severe depression (Croy et al. 2012a). In patients whose sense of smell improved during so-called “olfactory training”—a 12-week long, regular, intermittent exposure to a set of four odors (rose, eucalyptus, lemon, and cloves) (Hummel et al. 2009; Pieniak et al. 2022), relief of depressive symptoms has been observed (Sabiniewicz et al. 2022 but see: Pabel et al. 2020).

Two mutually non-exclusive pathways explain the mechanisms linking olfactory loss and depression (Croy et al. 2014). First, olfactory loss is associated with a loss of a source of pleasant sensations (Naudin et al. 2014; Parker et al. 2022), such as the appreciation of food. The enhanced social insecurity and loss of joy in the social aspects of eating may furthermore lead to social withdrawal, potentially accelerated by worries about own profession. This detriment in olfaction-related quality of life can increase depressive symptoms (Liu, Prem, Sharma, et al. 2022a). Second, altered brain functioning after olfactory loss may affect emotion processing. Animal studies, for instance, showed that olfactory loss reduces projections toward the amygdala (Carlsen et al. 1982; Mucignat-Caretta et al. 2004)—one of the major emotion-processing brain areas. In line, patients with olfactory loss assess emotional pictures as less salient and show diminished brain responses when viewing them (Han et al. 2019). Interestingly, the size of the olfactory bulb relates inversely to depression (Negoias et al. 2010) and even predicts the success of psychotherapy (Negoias et al. 2016). In some aetiologies of olfactory disorder, such as CRS, proinflammatory cytokines IL-6 and TNF-α may cross the blood-brain barrier to affect the amygdala and promote emotional instability (Soler et al. 2015; Yuan and Slotnick, 2014). In line, respiratory diseases moderate the relationship between depression and olfaction (Pabel et al. 2018). Also, other mental disorders, such as schizophrenia, are related to olfactory disorders (Moberg et al. 2014).

Olfactory loss affects quality of life less than blindness or deafness (Fischer et al. 2009) but is somewhat related to psychological distress (Bochicchio et al. 2023), depression (Croy and Hummel, 2017; Eliyan et al. 2021; Kamrava et al. 2021), and loneliness (Sivam et al. 2016). This relation is mediated by the temporal decline of olfaction, and patients with sudden olfactory loss—as due to COVID-19—are on higher risk for psychological distress (Kim et al. 2024). Altered behaviors in the areas affected by the olfactory disorders, along with the feeling of being lost and surprised by the sudden smell disorder, often result in the feeling of not being fully understood by close family members and friends (Burges Watson et al. 2021). Without the sense of smell, friends and romantic relationships are more difficult to initiate and maintain (Mahmut and Croy, 2019; Blomkvist and Hofer, 2021). Anosmia is related to a less satisfying sex life, especially for men (Schäfer et al. 2019; Deng et al. 2020; Siegel et al. 2021) and in people in stable romantic relationships (Hofer et al. 2025).

Olfactory loss imposes anxiety (Lemogne et al. 2015; Schäfer et al. 2019; Drummond et al. 2024). One of the spheres reflecting elevated anxiety is social chemical communication. The inability to smell hinders control over one’s body odor and raises concerns about the perception by others (Blomqvist et al. 2004; Nordin et al. 2011; Boesveldt and Parma, 2021). For the smell-disordered parents, these worries extrapolate to children (Croy et al. 2014; Lee et al. 2024). Consequently, patients suffering from smell loss may exaggerate personal hygiene routines by washing themselves several times a day or overdosing on scented cosmetics to mask the potential occurrence of unpleasant body odor (Miwa et al. 2001; Temmel et al. 2002b). Some of them withdraw from social events to avoid being singled out by body odor (Pellegrino et al. 2021).

Professional life may be negatively impacted by the loss of the sense of smell. 3-8% of the patients complain about their work performance after losing the sense of smell (Blomqvist et al. 2004; Nordin et al. 2011). Occupations particularly affected by this disorder are care professions, such as nurses and nursery teachers but also firefighters, chefs, perfumers, and sommeliers. Up to 60% of patients report the need to adjust their professional position to their condition, while 5% report the need to shift careers (Haxel et al. 2012).

#### Parosmia and phantosmia

Patients with qualitative olfactory disorders admit they get disgusted by their partner’s smell and avoid sharing this struggle with their partner so as not to hurt them (Burges Watson et al. 2021). In extreme cases, patients report for instance “quitting dating due to the lack of control over their body odor and the inability to imagine how a potential partner smells” or “altered feelings of intimacy due to the inability to smell the body odor of a partner or the partner’s body odor becoming disturbing” (Burges Watson et al. 2021). In their statements, patients with qualitative olfactory disorders often point out that they cannot share these feelings with their partner, as admitting that they are disgusted by their partner’s smell would hurt them.

### Consequences for personal safety and exposure to hazardous events

#### Anosmia and hyposmia

Olfactory loss exposes individuals to the risk of inhaling toxins and not detecting smoke or gas leaks. Dangerous events of potential poisoning happen to patients with anosmia 2 to 3 times more often than to people without olfactory impairment (Santos et al. 2004; Croy et al. 2012b; Pence et al. 2014; Coelho et al. 2021). Consequently, patients with smell disorders are anxious about staying home alone (Pellegrino et al. 2021; Lee et al. 2024). To cope with the difficulties in detecting environmental threats, patients with smell loss develop certain protective behaviors like specific attention not to leave the iron alone or to consult other household members about the freshness of the food products (Croy et al. 2012c; Elkholi et al. 2021). Increased alertness to facial expressions of fear, anger, and disgust has also been observed in anosmic individuals, concluding that they may be more vigilant to the social and visual cues for disgusting odors in the environment (Lemogne et al. 2015; Drummond et al. 2024).

### Measuring quality of life in olfactory disorders

Many studies concerning olfactory disorders concentrate on the general quality of life, which may sometimes be hard to examine from the perspective of olfactory disorders, as the questions do not tackle activities directly engaging the nose (Neuland et al. 2011). Several questionnaires dedicated to quality of life in olfactory disorders are available for different groups of patients (Han et al. 2020). An important development in understanding what aspects of the quality of life are affected by olfactory disorders is the involvement of the patients and the public. A summary of questionnaires on the quality of life associated with olfactory loss is presented in Table 1.

**Table 1.** Summary of available methods to measure general and olfaction-specific quality of life. Referenced methods appear in chronological order.

Reference	Questionnaire	Comment
(Ware and Sherbourne, 1992)	Short Form-36 Health Survey	General health survey
(Anderson et al. 1999)	Sinonasal Outcome Test (SNOT-16)	For patients with chronic rhinosinusitis (CRS), directly assesses olfactory dysfunction
(de Jong et al. 1999)	Appetite, hunger, subjective taste and smell questionnaire	Describes sensory impressions and feelings of appetite and hunger
(Miwa et al. 2001)	Questionnaire on the impact of olfactory impairment on quality of life and disability	Describes impairment in 15 olfactory-related daily life activities and general enjoyment of life
(Nordin et al. 2003)	Multi-Clinic Smell and Taste Questionnaire	Assessment of the consequences of olfactory dysfunction
(Frasnelli and Hummel 2005)	Questionnaire of Olfactory Dysfunction	Assessment of daily life problems associated with olfactory loss
(McDowell, 2006)	General Well-Being Schedule	Contains positive and negative questions across six dimensions: well-being, self-control, vitality, depression, anxiety, and general health
(Croy et al. 2010)	Individual significance of olfaction (ISoO)	Addresses associations, applications, and consequences of olfaction in daily life
(Pusswald et al. 2012)	Brief Self-Report Inventory to Measure Olfactory Dysfunction and Quality of Life	Assessment of the subjective general and odor-specific olfactory function and olfaction-related quality of life
(Mattos et al. 2018)	Questionnaire of Olfactory Dysfunction—Negative Statements (QOD-NS)	QOD-NS describes the consequences of olfactory loss. Subscale of Positive Statements (QOD-PS) is considered a measure of how well a patient is coping with the olfactory disorder (Liu et al. 2022b)
(Lee et al. 2022)	Olfactory Dysfunction Outcomes Rating (ODOR)	Includes questions about the consequences of olfactory dysfunction

### Dysosmia-related quality of life across the lifespan

#### Children and adolescents

Our understanding of the prevalence of olfactory disorders in the pediatric population and their consequences for the children is still far from complete. Despite the increasing evidence for high olfactory abilities in children and functionality of the olfactory system since birth (Schaal 1988, 2000; Soussignan and Schaal, 1996; Stevenson et al. 2007; Gellrich et al. 2017; Oleszkiewicz et al. 2022; Ustun et al. 2022), we poorly understand the impact odors have on children and whether olfactory loss has implications on their quality of life. Since children and adolescents are a minority of patients with olfactory loss referred to ENT clinics (2% to 4% of all patients), it can be expected that olfactory loss is less prevalent in the pediatric population—but may also go unnoticed (Gellrich et al. 2025). It is plausible that children adjust to the smell disorder quickly and do not report problems with olfaction to their parents or healthcare professionals. To our knowledge, this assumption has not been tested empirically. More studies involving the perspective of children and adolescents suffering from olfactory loss are needed to understand the consequences of olfactory disorders in their daily lives, including family bonding, relationships with peers, and effects on emotional and cognitive functioning.

#### Adults

Olfactory disorders are most salient for the subgroup of people who are used to very good olfactory function—young and healthy individuals—and for the subgroup who value their sense of smell highly—younger women (Murr et al. 2018). Indeed, younger patients with olfactory loss and women report lower olfaction-related quality of life (Zou et al. 2021). Sudden loss of the ability to perceive odors is harder to adapt to in comparison to congenital anosmia (Romanowicz et al. 2022). This is particularly evident among adults who have suddenly lost their sense of smell due to COVID-19 (Coelho et al. 2021; Elkholi et al. 2021; Bochicchio et al. 2023; Hofer et al. 2025).

In contrast, some people practically cannot smell, but they still consider their olfaction normal and do not notice the deficit. Unawareness of olfactory loss is mostly driven by age and often goes unnoticed under the cover of other emerging health conditions (Oleszkiewicz and Hummel, 2019). People with undetected olfactory loss, who have not been recruited in the ENT clinic, report similar quality of life, and depressive symptoms as individuals with normosmia, but exhibit slightly lower cognitive capacities possibly related to the association between olfactory loss and cognitive dysfunction (Oleszkiewicz et al. 2019).

#### Older adults

Older individuals typically report that their vision and audition declined considerably as compared to when they were young—however, they also report an unaffected sense of smell (Cavazzana et al. 2018). This is surprising as age is the number one cause for quantitative olfactory disorders. This result exemplifies how little attention slowly evolving olfactory disorders receive. In line, people with sudden olfactory loss, as in viral or traumatic cases, report higher impairment (Zou et al. 2021). Due to the gradual development, olfactory deficits may go unnoticed, exposing elderly people to environmental hazards and household accidents related to respiratory intoxication or food poisoning (Stevenson, 2010; Croy et al. 2014). Among people older than 70 years, 20% to 31% cannot detect odors of smoke and natural gas (Hoffman et al. 2016). Olfactory disorders emerging with aging may contribute to poor diet (Toussaint et al. 2015), especially in women presenting moderate/severe olfactory impairment (Gopinath et al. 2016). In the geriatric population, olfactory loss has also been linked to lesser variability of chosen foods (Rolls and McDermott, 1991; Kremer et al. 2014). Consequences of olfactory loss of social functioning, and depression are also amplified with aging (Eliyan et al. 2021). Olfactory loss in aging individuals comes with a smaller social network (Zou et al. 2016; Boesveldt et al. 2017), and loneliness (Sivam et al. 2016; Desiato et al. 2021). Olfactory loss is being considered a mortality risk marker in older individuals due to its relationship with increased frailty, neurodegeneration, poor nutrition, and inflated risk of being exposed to life-threatening situations (Pinto et al. 2017; Van Regemorter et al. 2020; Ruane et al. 2025).

### Treatment strategies and coping with the olfactory disorders

As we already concluded in previous reviews about QoL in olfactory disorders and found confirmed with updated literature from the last decade, loss of the sense of smell negatively impacts quality of life, exposes patients with anosmia or hyposmia to environmental hazards, devoid them of eating pleasures, and hinders social interactions (Hummel and Nordin 2005; Croy et al. 2014).

Olfactory disorders are not irreversible; multiple treatment options exist (Whitcroft et al. 2023). Olfactory training has been demonstrated to be an efficient treatment method in multiple olfactory loss etiologies, including post-traumatic and post-infectious olfactory loss (Hummel et al. 2017; Whitcroft et al. 2023). Olfactory training can be recommended to individuals with lower baseline scores, but who have some degree of olfactory function (to sense the odorants they sniff bidaily). Individuals who began regaining olfactory function and manifesting parosmia are also likely to benefit from olfactory training (Liu et al. 2021). Besides the sense of smell, olfactory training may benefit cognitive and emotional functions (Pieniak et al. 2024), especially in the geriatric population (Wegener et al. 2018; Oleszkiewicz et al. 2021; Oleszkiewicz et al. 2021; Woo et al. 2023; Vance et al. 2024) which ultimately should improve quality of life. Still, many patients remain unaware of such treatment possibility (Li et al. 2024). Patients who reported the greatest reduction in the quality of life because of olfactory disorders are most motivated to perform an olfactory training regimen, but the lack of noticeable improvement within a short period of time is the main reason patients drop the procedure after approximately one month (Li et al. 2024). Thus, medical recommendations for olfactory training should be accompanied by a clear message that the method is only effective when performed regularly over at least 12 weeks.

Pharmacological treatment is also available to patients suffering from olfactory loss. While awaiting rigorous examination in appropriate double-blind, multicentric investigations (Patel et al. 2022), preliminary evidence shows therapeutic effects in olfactory dysfunction due to various causes, e.g. sodium citrate (Whitcroft et al. 2017, 2021), topical vitamin A (Reden et al. 2012; Hummel et al. 2017), zinc (Lyckholm et al. 2012; Jiang et al. 2015), or acupuncture (Drews et al. 2021). In addition, intranasal and systemic corticosteroids, surgery, or monoclonal antibodies are recommended for patients with olfactory loss resulting from sinunasal disease (Whitcroft and Hummel, 2019).

In addition to these forms of treatment, which have been used for years, sometimes with limited effectiveness (Patel et al. 2022), several new therapeutic options are currently being investigated (Mainland et al. 2020). They include injections of platelet-rich plasma into the olfactory cleft (Yan et al. 2023) or the topical administration of theophylline. Other work is currently underway on the effects of transcutaneous electrical stimulation to augment smell training (Maharjan et al. 2018). More futuristic aspects of therapy (Gunder et al. 2023) include work on olfactory implants (Lipp et al. 2025)—in analogy to cochlear implants—or transplantation of olfactory mucosa (Kurtenbach et al. 2019).

Patient survey data, however, showed that most frequently used treatment options—nasal and oral steroids and smell training—are perceived as only slightly or not effective at all by the majority of participants in the survey. Younger age thereby seemed the main predictor of treatment success (Murphy et al. 2024). Treatment options should hence be tailored to the patient’s age and also to the cause of olfactory loss to ensure maximal efficiency, and more research is needed to better target mechanisms for chemosensory impairment.

Most individuals adapt to the chemosensory deficit, and their quality of life improves again with the duration of the dysfunction (Auinger et al. 2021; Liu et al. 2022), while the importance of olfaction decreases (Liu et al. 2020). Coping strategies can potentially be guided during psychotherapy, which has been proven to be a successful intervention to reduce anxiety and depression in individuals suffering from sensory impairments in visual and auditory domains (Trott et al. 2025). Empirical evidence regarding olfactory dysfunctions is needed.

Support groups may also turn out helpful when adjusting to olfactory loss. There are non-governmental organizations (NGOs) dedicated to olfactory disorders (e.g. Chrissi Kelly on Smell [CKOS], Smell and Taste Association of North America [STANA], Fifth Sense, or Reuksmaakstoornis), or a recently opened NIH National Smell and Taste Center that combines research and treatment of olfactory disorders. These organizations create forums for patients to seek peer support, promote the inclusion of patient voices in the treatment process and research on olfactory dysfunction, advocate for funding aimed at better understanding olfactory disorders and treating them, and build networks connecting patients, healthcare professionals, and institutions to educate about olfactory health and disease. Patients and public involvement appear critical for the development of research, social strategies, clinical approaches, and science aimed at understanding olfactory disorders (Gane et al. 2020; Parker et al. 2021a, 2021b, 2022; Smith et al. 2021).

## Data Availability

No new data were generated or analysed in support of this research.
